# Genetic Differences between Male and Female Pattern Hair Loss in a Korean Population

**DOI:** 10.3390/life14080939

**Published:** 2024-07-26

**Authors:** Jihyun Lee, Ja-Eun Choi, Joohun Ha, Youngjoo Kim, Changhyun Lee, Kyung-Won Hong

**Affiliations:** 1 Easy Hydrogen Corporation, Jeju City 63196, Republic of Korea; easyhydrogen@gmail.com; 2 Institute of Advanced Technology, Theragen Health Co., Ltd., Seongnam 13493, Republic of Korea; jaeun.choi@theragenhealth.com; 3 Department of Biochemistry and Molecular Biology, Graduate School, College of Medicine, Kyung Hee University, Seoul 02447, Republic of Korea; hajh@khu.ac.kr; 4 Department of Urology, College of Medicine, Jeju National University, Jeju City 63243, Republic of Korea; kurology@naver.com; 5 Chunjieh Cooperation, Jeju City 63359, Republic of Korea; 7771rkstlr@hanmail.com

**Keywords:** androgenetic alopecia, female pattern hair loss, genome-wide association study, TSNARE1, FZD1

## Abstract

Studies on androgenetic alopecia (AGA or patterned hair loss (PHL)) have suggested different underlying pathological mechanisms between males and females. While many genetic factors for male hair loss have been identified through genome-wide association studies (GWASs), the genetic determinants of female hair loss remain unclear. In this study, we analyzed approximately 1000 individuals (436 males and 568 females) to identify sex-specific genetic factors. We conducted three independent GWASs for the total, male-only, and female-only groups, identifying three novel loci (rs7814359, rs2163085, and rs4793158 of the TSNARE1, FZD1, and GJC1 genes, respectively). rs7814359 showed a significant genome-wide association with AGA in the combined sex group and a weak association in both the male-only and female-only groups. The single nucleotide polymorphism (SNP) rs2163085 showed a significant genome-wide association with AGA in the combined group and notable significance in females. The rs4793158 SNP showed a suggestive association with AGA in both the combined and female-only groups. TSNARE1, related to rs7814359, is involved in vesicle transport. FZD1 is a key regulator of the Wnt signaling pathway. GJC1 is a gap junction protein. The associations of FZD1 and GJC1 with female-specific AGA suggest that sex hormones, such as estrogen, may influence FPHL through these genes. These findings will contribute to our understanding of the sex-specific pathophysiology of AGA.

## 1. Introduction

In 1960, Orentreich established the term “androgenetic alopecia” (AGA) which is also known as “male pattern baldness”, “common baldness”, “male pattern alopecia”, and “male pattern hair loss” (MPHL) [1]. Although AGA can be understood as a symptom of hair loss mediated by systemic androgens and genetic factors primarily in men, it also broadly covers pattern hair loss occurring in women [1]. According to medical health statistics provided by the Health Insurance Review and Assessment Service (HIRA) of Korea in 2021 (https://opendata.hira.or.kr (accessed on 11 January 2024)), a significant proportion (44%) of the 242,960 patients diagnosed with AGA are women [2,3]. This result indicates that studying female hair loss is as important as male hair loss [2,3].

MPHL and female pattern hair loss (FPHL) share similar underlying causes and histopathological features but display distinct morphological characteristics [4,5,6,7,8]. MPHL is primarily driven by genetic factors related to the androgen pathway, resulting in a characteristic hair loss pattern, starting at the frontal hairline and receding backward to form an “M” shape, accompanied by vertex thinning [9,10]. Over time, the hair becomes finer and less dense, creating a U-shaped pattern of hair loss around the sides of the head and a bald spot at the back [9,10]. Conversely, FPHL was recognized as environmental or multifactorial disease with fewer genetic factors than MPHL [3,11,12,13]. Some studies have indicated that FPHL may occur even in the absence of androgens [14,15,16]. Typically, FPHL presents as a general reduction in hair density, particularly affecting the mid- and frontal scalp regions while preserving the frontal hairline, forming a characteristic “Christmas tree” baldness pattern [17,18,19]. Genome-wide association studies (GWASs) are the most common genomic analyses for discovering genetic factors for a specific phenotype. To date, more than a dozen GWAS papers have been published on MPHL, and candidate genetic factors related to the sex-hormone pathway have been reported [20]. While numerous genetic factors have been identified for male hair loss, the genetic determinants of female hair loss remain unclear [20]. However, these studies suggest that the underlying pathological mechanisms between male and female hair loss have differences [20].

**Figure 1:**
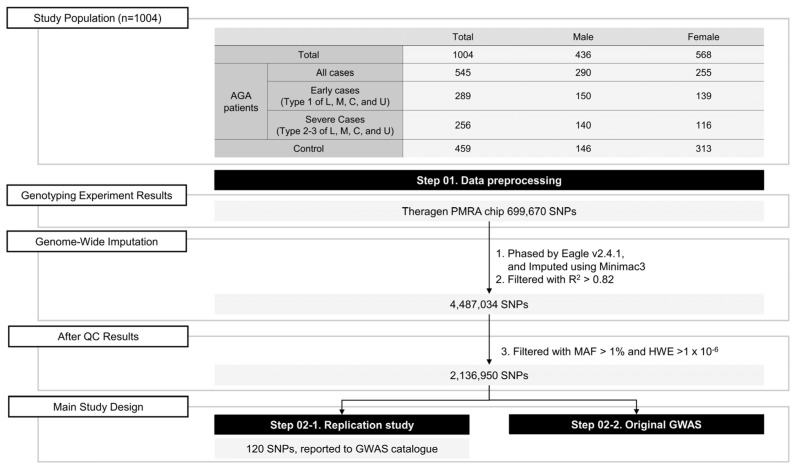
Study design.

In this study, we identified sex-specific genetic factors in approximately 1000 individuals, including 436 males and 568 females, and provided valuable insights into the broader field of hair loss genetics. The study design is illustrated in Figure 1.

## 2. Materials and Methods

### 2.1. Study Participants

The study population was recruited from Lee Jihyun Clinic in South Korea, consisting of 1004 individuals; among them, 545 had AGA (290 males and 255 females). The remaining 459 individuals, including 146 males and 313 females, formed the control group. The study was conducted in accordance with the Declaration of Helsinki and approved by the Korean Skin Research Center Institutional Review Board (IRB No. HBABN01-230320-BR-E0007-01). Written informed consent was obtained from all the participants. The overall study design is schematically illustrated in Figure 1.

According to the basic and specific (BASP) classification, the shape of the anterior hairline was classified into one of four basic types (L1–3, M1–3, C1–3, or U1–3), and the specific types (V1–3 or F1–3) were classified according to mid-scalp hair density. The BASP classification of hair loss was used to diagnose participants with M2, C1, or U1 (basic type) or V1 or F1 (specific type) AGA. In this study, we aimed to analyze the genetic impact on the detailed types of BASP classification. We categorized each of the L, M, C, and U types into two groups: type 1 as early hair loss and types 2–3 as severe hair loss (see Figure S1). We then conducted a further analysis on the major genetic factors identified in the comprehensive case–control study.

The age of the control group individuals was 50 years or older, they had no family history of AGA, and were categorized as L or M0 (basic type) and V0 or F0 (specific type) according to the BASP classification. A single dermatologist carefully evaluated all the participants for hair loss status and family history to ensure accurate data analysis. No participant was included in previous studies.

### 2.2. Genotyping

DNA was extracted from blood samples and subsequently amplified and randomly fragmented. The resulting 25–125 bp fragments were purified, resuspended, and hybridized with a Theragen Precision Medicine Research Array (Theragen PMRA array), which is a customized platform based on the Asian Precision Medicine Research Array (Thermo Fisher Scientific, Waltham, MA, USA).

Following hybridization, stringent conditions were applied to wash the bound targets, to eliminate nonspecific background signals and minimize noise from random ligation events. Genotyping was conducted using the Theragen PMRA array, as per manufacturer’s instructions, facilitating the assessment of 699,670 single nucleotide polymorphisms (SNPs).

The Theragen PMRA array provides comprehensive genome-wide coverage of five major populations. All samples were subjected to rigorous quality control measures to ensure data quality. The criteria of a dish quality control exceeding 0.82 and a sample call rate surpassing 0.95 were employed to guarantee the reliability and accuracy of the genetic data obtained from the genotyping process.

### 2.3. Imputation and Quality Control

Pre-phasing was performed using Eagle v2.4.1, and doses were imputed using minimac3 and the 1000 Genomes Project Phase 1 (version 3) East Asian reference haplotypes to enhance genotyping accuracy. A total of 4,487,034 SNPs were imputed, with the imputation r^2^ > 0.8. The inclusion criteria included genotype call rates (≥0.99), minor allele frequency (≥0.01), Hardy–Weinberg equilibrium *p*-value > 1 × 10^−6^. In this study, 2,136,950 SNPs were used.

### 2.4. Literature Survey

To identify previously reported AGA SNPs, the keywords “Androgenetic Alopecia”, “Male-pattern baldness”, “Male-pattern hair loss”, and “Pattern hair loss” were searched in the GWAS catalog site (https://www.ebi.ac.uk/gwas/ (accessed date 11 June 2024)), the most popular database on GWAS reports [21]. In total, 120 SNPs were selected for AGA replication studies. These SNPs were associated with AGA across eight distinct cohorts, including seven studies focusing on individuals of European ancestry and one study on Koreans.

### 2.5. Statistics and Software

For GWASs, genetic association analyses were conducted via dividing the collected samples into three groups: all-samples, male-only, and female-only groups. In this study, we conducted replication studies for previously reported AGA SNPs and the original genome-wide association studies in each group. The individuals with AGA cases and non-AGA control groups were coded as case (1) and control (0), and logistic regression analysis was performed via controlling for age and sex (for total sex group) and for age (for male-only and female-only groups) as covariates. The significance of the replication study was defined as satisfying the following criteria: having a consistent risk trend for AGA with a *p*-value < 0.05 and exhibiting a more significant odds ratio (OR) or *p*-value compared to previously reported studies. To conduct high-throughput analyses, the popular GWAS software PLINK version 1.9.0 was used. A *p*-value < 5 × 10^−8^ was applied as the genome-wide significant *p*-value criterion, and a *p*-value < 1 × 10^−5^ was applied as the genome-wide suggestive association *p*-value criterion.

To depict the GWAS results, a Manhattan plot was generated at the genome level using R (version 4.1.2; https://cran.r-project.org/bin/windows/base/ (accessed date 11 June 2024)), and a signal plot was generated to zoom in on the highlighted association using LocusZoom (version 0.4.8.2) [22]. The expression quantitative plots were obtained from the GTEX portal (https://www.gtexportal.org/home/ (accessed date 11 June 2024)) to understand the lead SNP functional importance [23].

## 3. Results

### 3.1. Population Characteristics

This study included 1004 individuals living in Jeju Island, South Korea, of which 436 were male (43.4%) and 568 were female (56.6%) (Table 1). Among these individuals, patients with AGA (n = 545, age 46.7 ± 14.6 years) were diagnosed with hair loss at a clinic (Lee Jihyun Clinic, Jeju, Korea). Non-AGA controls (n = 459) demonstrated no hair loss symptoms and were older than 50 years (53.2 ± 7.1 years). The male-only group consisted of 290 patients with AGA and 146 without AGA, whereas the female-only group consisted of 255 patients with AGA and 313 without AGA. Within the male group, the proportion of individuals with AGA was higher at 66.5% compared to the female group. The average age of individuals with hair loss was less than 50 years in both male and female groups, whereas those without AGA were older than 50 years. When the case group was classified into early cases and severe cases, the proportion of early cases was found to be slightly higher.

### 3.2. Recapitulation Study

In this study, we obtained recapitulation target SNPs from 11 GWASs, including 120 SNPs from the GWAS catalog (see Table S1). Table S1 describes the results for the three groups (total: PHL group, male-only: MPHL group, and female-only: FPHL group). We defined the replicated SNPs as having the same effect direction of OR or beta value, with a *p*-value < 0.05, in at least one testing group among PHL, MPHL, and FPHL. Among the tested SNPs, eight SNPs (rs9282858, rs3827760, rs201563, rs2073963, rs6047844, rs1160312, rs10888690, and rs13021718) showed the same direction (*p* < 0.05) in PHL (Table 2). Among these, four (rs201563, rs6047844, rs1160312, and rs7976269) were more significant in MPHL, three (rs9282858, rs3827760, and rs2073963) were more significant in FPHL, and the remaining one SNP (rs13021718) had similar significance in all groups. We further conducted a meta-analysis of the previously reported results and the current study results, which revealed that seven of the eight SNPs showed enhanced significance in the meta-analysis *p*-values (see underlined results in Table 2).

For the rs92858 (SRD5A2) and rs2073963 (HDAC9) genes, significant associations were observed in the female group regardless of BASP type. The rs3827760 (EDAR) gene showed a stronger association with the early cases. The rs201563 (PAX1), rs6047844 (LINC01432), rs1160312 (LINC01432), and rs13021718 (DPY30) genes were more significantly associated with the severe cases. The rs10888690 (FAF1) gene was associated with overall hair loss regardless of BASP type or sex. The rs7976269 (FAR2) gene showed a more significant association with the severe cases, particularly in the male group.

### 3.3. GWAS

**Figure 2:**
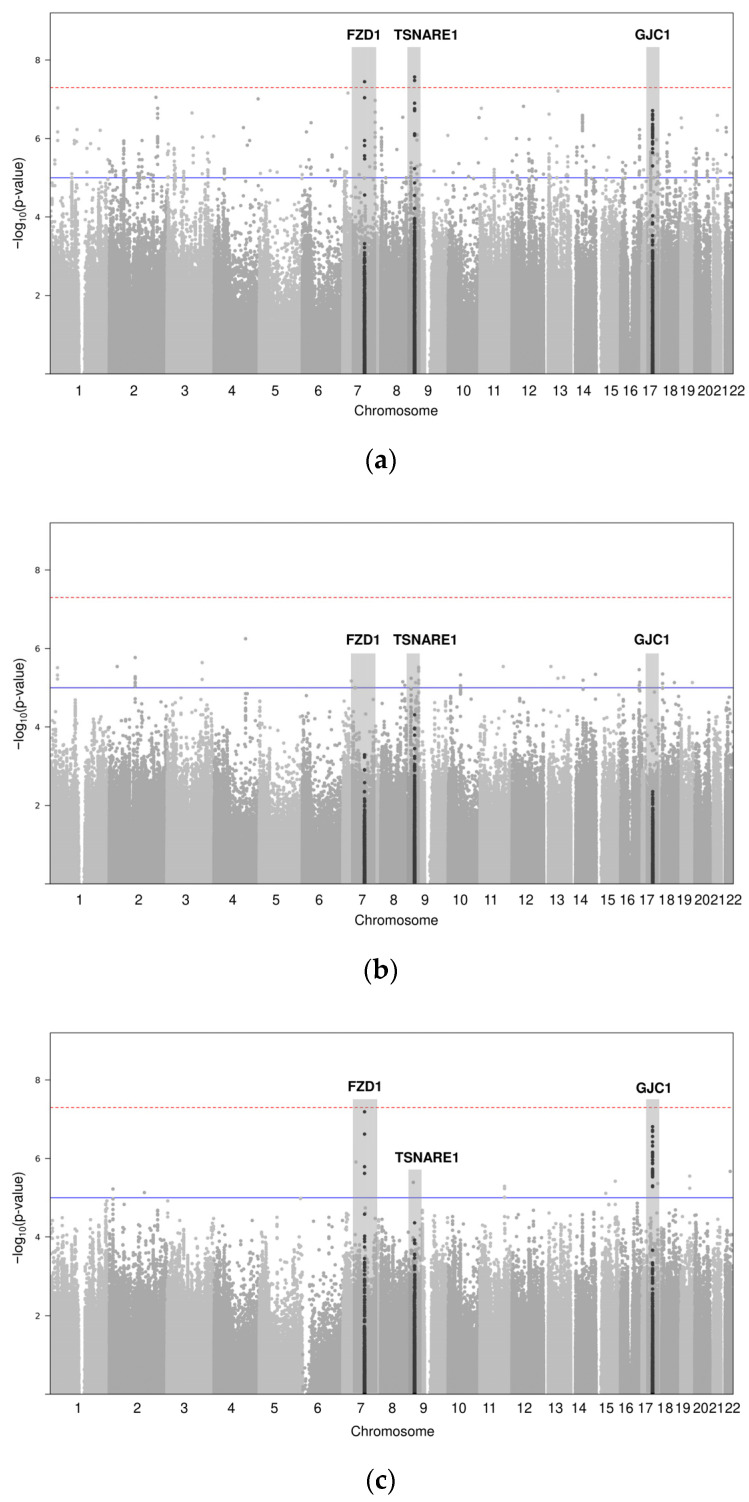
Manhattan plots. As a result of the GWAS, the *p*-values were transformed to −log10 and plotted on each chromosome position. The dotted line is the criteria of the significant association *p*-value (5 × 10^−8^), and the solid line is the criteria of the suggestive association *p*-value (1 × 10^−5^). The gray highlighted part indicated the area of the new hair loss genetic index that was mainly discovered in this study. (**a**) All-samples group; (**b**) male-only group; (**c**) female-only.

**Figure 3:**
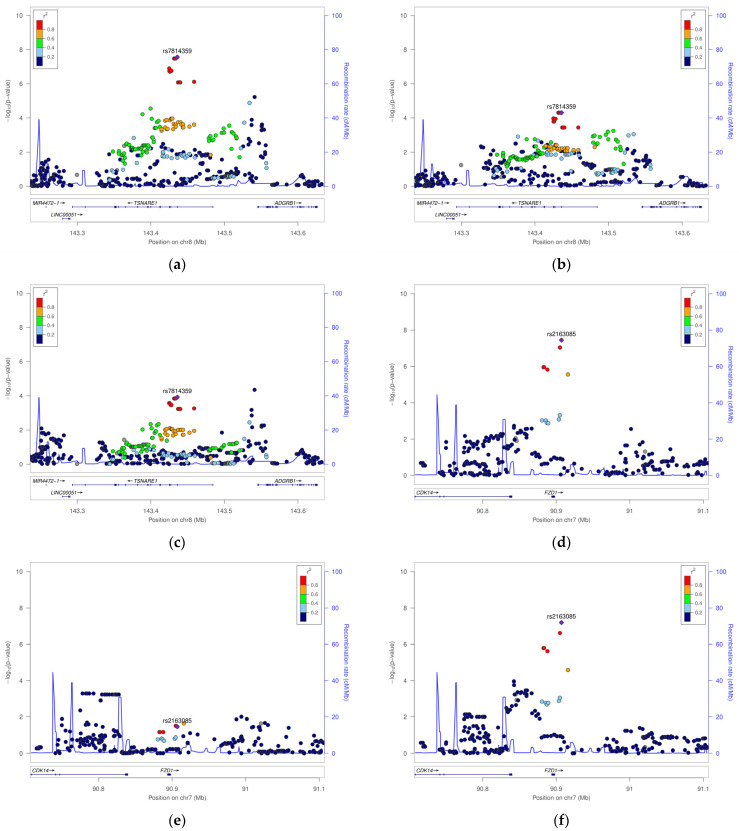

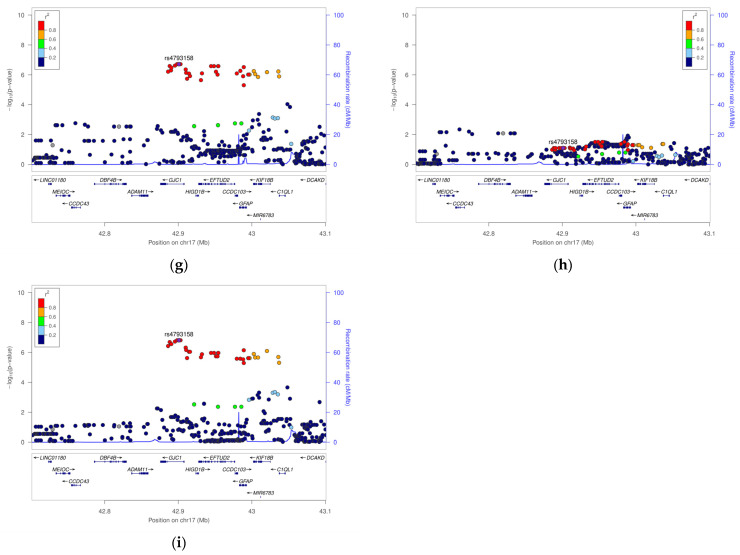
Signal plots. Graphical illustration of the SNP ± 400 kbp position and mapped genes discovered in the GWASs. (**a**) rs7814359 (TSNARE1) of all-samples group; (**b**) rs7814359 of male-only group; (**c**) rs7814359 of female-only group; (**d**) rs2163085 (FZD1) of all-samples group; (**e**) rs2163085 of male-only group; (**f**) rs2163085 of female-only group; (**g**). rs47933158 (GJC1) of all-samples group; (**h**) rs4793158 of male-only group; (**i**) rs4793158 of female-only group.

Association analyses were extended to the genome level to understand population-specific genetic factors. The GWAS results are shown in Figure 2. Figure 2a shows the PHL group results highlighted by black dots for two genome-wide significant SNPs (rs2163085 and rs7814359 of FZD1 and TSNARE1 gene loci, respectively) and one suggestive association SNP (rs4793158 of the GJC1 gene locus). Figure 2b shows the results for the MPHL group; no significant SNP was observed. Figure 2c shows the FPHL group results, wherein one genome-wide significant SNP (rs2163085 of FZD1) and one suggestive association SNP (rs4793158 of GJC1) were detected. Figure 3 illustrates the top three SNP regions using signal plots.

Descriptions of the association results are provided in Table 3. The individuals with G allele genotypes of rs7814359 showed significant protective effects on hair loss (*p*-value = 2.7 × 10^−8^, OR = 0.57, 95% confidence interval, CI: 0.46–0.69) in the PHL group. This effect was constant but not significant in MPHL (*p*-value = 4.9 × 10^−5^, OR = 0.53, 95% CI: 0.39–0.72) and in FPHL (*p*-value = 1.2 × 10^−4^, OR = 0.60, 95% CI: 0.46–0.78). The SNP rs2163085 showed that individuals with C allele genotypes revealed a significant risk of hair loss (*p*-value = 3.6 × 10^−8^, OR = 1.88, CI: 1.50–2.35) in the PHL group, and this association was only significant in the FPHL group (*p*-value = 6.4 × 10^−8^, OR = 2.24, CI: 1.67–3.01).

In addition, we identified a suggestive association with the GJC1 intronic region. In the all-samples group, individuals with C allele genotypes of rs4793158 revealed an increased risk of AGA (*p*-value = 1.9 × 10^−7^, OR = 2.15, CI: 1.61–2.87) in PHL, and this association was significant only in the FPHL group (*p*-value = 1.5 × 10^−7^, OR = 2.70, CI: 1.86–3.91).

For the rs7814359 (TSNARE1) gene, significant associations were observed in all test groups regardless of BASP type. The rs2163085 (FZD1) and rs4793158 (EFTUD2) genes showed significance in the female group regardless of BASP type.

## 4. Discussion

We conducted a recapitulation study for the previously reported AGA GWAS SNPs and population-specific GWASs in the three groups. In this study, we confirmed the replication of well-established AGA genetic factors and identified three novel genetic factors in a sex-specific manner.

The replicated SNPs were rs9282858 of the SRD5A2 gene, rs3827760 of the EDAR gene, rs201563 of the PAX1 gene, rs2073963 of the HDAC9 gene, rs6047844 and rs1160312 of LINC01432, rs10888690 of the FAF1 gene, rs13021718 of the DPY30 gene, and rs7976269 of the FAR2 gene. SNPs located in the 20p11.3 (PAX1), HDAC9, and EDAR genes were previously identified as significant genetic factors for AGA, particularly in men of European ancestry; our study validated them in a Korean population. SRD5A2 and EDAR were replicated in the female-only group, and 20p11.3 (PAX1) was replicated in the male-only group. These results suggest that the replicated markers identified in our study are genetic factors associated with hair loss across all ethnicities and that hair loss may be a disease modulated by sex hormones.

GWASs for the three groups (PHL, MPHL, and FPHL) revealed three novel genetic loci, rs7814359 (TSNARE1), rs2163085 (FZD1), and rs4793158 (GJC1). Of these, rs7814359 is located in the TSNARE1 coding region, and its association tendency was similar among all three groups; rs2163085 was located on the 3′ flanking region of a Wnt signaling pathway gene (termed as FZD1), and the association was specific only in the FPHL group; rs4793158 was located in the intron region of the gap junction protein gene (GJC1), and this association was specific only to the FPHL group.

The TSNARE1 gene encodes the T-SNARE Domain Containing 1 protein, which forms a SNARE complex with the soluble N-ethylmaleimide-sensitive factor (SNAP) attachment protein and is involved in exocytosis [24,25,26,27]. The most significant SNP (rs7814359) showed increased expression of quantitative trait loci (eQTLs) in the skin, brain, and arteries on the GTEX portal (https://www.gtexportal.org/home/ (access date 11 June 2024)) [23] (Figure S2A). Therefore, our results suggest that the SNAP pathway is widely expressed in hair follicles and influences hair-linked extracellular factors [25,27,28,29,30,31]. The absorption of extracellular growth factors may enhance the growth rate of hair follicles, potentially accelerating the hair growth cycle and contributing to hair loss [32].

The FPHL-specific genetic factor (FZD1 gene) encodes the frizzled receptor for the Wnt signaling protein. The Wnt signaling pathway is crucial for regulating the hair growth cycle, activating hair follicle cell development, and promoting hair regeneration [33,34,35,36]. A study has suggested that the Wnt signaling pathway influences FPHL [20]. According to this study, changing the expression of the Wnt signaling pathway gene DKK-1 enhances cell proliferation and transformation, with increased expression in patients with hair loss [20]. DKK-1 has been reported to induce hair loss, showing increased expression in the dermal papilla cells with advanced hair loss [20]. Furthermore, the Wnt signaling agonist R-spondin 1 (RSPO1) acts as an anti-agonizing factor of DKK-1 to improve the condition of hair [20]. Estrogen, a female hormone, promotes the expression of frizzled genes involved in Wnt signaling [37]. After menopause, which is characterized by a decline in estrogen from the ovaries, the prevalence of FPHL exceeds up to 50% in women [3,38,39,40]. These results suggest that the Wnt signaling pathway is associated with FPHL through a decline in estrogen levels. Some studies have reported that FPHL may occur even in the absence of androgens [14,15,16]

GJC1 is a connexin gene that encodes a gap junction component protein that plays an important role in cell-to-cell connections and is essential for cellular interaction and structural stability [41,42,43]. These functions contribute to maintaining stable hair fiber morphology and promoting hair growth [42,43]. Connexin is known to be required for female reproductive functions, and its loss causes an oocyte deficiency [44,45]. Oocytes play a role in regulating the development of ovarian mural granulosa cells, which produce estrogen [46]. This suggests that abnormalities in connexin levels may be related to estrogen levels and that the Wnt signaling pathway, regulated by estrogen levels, along with abnormalities in hair follicle cell structure, is significantly associated with FPHL.

The primary mechanisms of hair loss involve abnormalities in cell-to-cell adhesion structures, signal transmission pathways, and hair follicle growth cycle regulation [47,48,49,50]. In this study, we observed significant associations with genes related to these mechanisms. These results may support the existing understanding of hair loss mechanisms and suggest a genetic influence that varies according to sex hormone levels.

This study has several limitations. First, the results were based on statistical analyses, suggesting potential associations with hair loss; however, identifying this function through in vitro or in vivo experiments may be necessary. Second, the study sample size was approximately 1000, which is relatively small compared to larger-scale GWASs conducted abroad. Therefore, further studies are necessary to validate and replicate the genes identified in this study. Third, the study participants were from isolated regions of Jeju Island; hence, determining the replication of the results in other populations is necessary. However, based on the consistency of the markers reported in previous studies and their similar trends in this study, the case/control definition of patients with hair loss or the specificity of the study group is unlikely to have significantly affected the findings.

## 5. Conclusions

We found that genes previously associated with hair loss in men had a similar significance in women. Furthermore, our study suggests that the mechanism of transportation of substances inside and outside cells could impact the hair growth cycle in both sexes, potentially leading to hair loss. Additionally, our findings indicate that FPHL could originate from disruptions in Wnt signaling and hair cell instability. These results advance our understanding of the biological mechanisms underlying hair loss and suggest targets for personalized hair loss treatment tailored to specific sexes.

## Figures and Tables

**Table 1:** Population characteristics.

		All Subjects (n = 1004)		Males (n = 436, 43.4%)		Females (n = 568, 56.6%)	
		n (% of All)	Age (Mean ± s.d)	n (% of Males)	Age (Mean ± s.d)	n (% of Females)	Age (Mean ± s.d)
AGA cases	All cases	545 (54.3)	46.7 ± 14.6	290 (66.5)	45.4 ± 14.9	255 (44.9)	48.2 ± 14.1
	Early cases	289 (28.8)	42.4 ± 13.8	150 (34.4)	39.6 ± 14.0	139 (24.5)	45.5 ± 14.0
	Severe cases	256 (25.5)	51.5 ± 14.0	140 (32.1)	51.5 ± 14.0	116 (20.4)	51.5 ± 14.0
Control		459 (45.7)	53.2 ± 7.1	146(33.5)	52.9 ± 7.1	313 (55.1)	53.3 ± 7.2

Note: AGA, Androgenetic alopecia; early cases, Early hair loss type (type 1 group of each of BASP classification L, M, C, and U); severe cases, Severe hair loss type (type 2–3 group of each of L, M, C, and U).

**Table 2:** Replicated SNPs in the present study among the previously reported SNPs.

SNP	Mapped Gene	Function	A1	Case Type	Present Study						Previous Study		Meta-Analysis			
					All Subjects		Male		Female		OR	*p*	OR	*p*	Q	I
					OR (95%CI)	*p*	OR (95%CI)	*p*	OR (95%CI)	*p*						
rs9282858	SRD5A2	Missense	T	All	**0.28 (0.14–0.55)**	**2.3 × 10^−4^**	0.47 (0.20–1.10)	8.3 × 10^−2^	**0.10 (0.02–0.45)**	**2.5 × 10^−3^**	0.60	9.0 × 10^−18^	0.60	2.0 × 10^−17^	0.58	0
				Early	**0.31 (0.12–0.82)**	**1.8 × 10^−2^**	0.62 (0.19–2.09)	4.4 × 10^−1^	**0.10 (0.01–0.82)**	**3.1 × 10^−2^**						
				Severe	**0.31 (0.13–0.71)**	**6.0 × 10^−3^**	0.47 (0.17–1.28)	1.4 × 10^−1^	**0.11 (0.01–0.84)**	**3.3 × 10^−2^**						
rs3827760	EDAR	Missense	A	All	**0.62 (0.47–0.81)**	**6.3 × 10^−4^**	0.73 (0.48–1.10)	1.4 × 10^−1^	**0.54 (0.37–0.79)**	**1.4 × 10^−3^**	0.66	1.0 × 10^−14^	0.67	3.4 × 10^−16^	0.66	0
				Early	**0.46 (0.31–0.68)**	**9.0 × 10^−5^**	**0.54 (0.30–1.00)**	**4.8 × 10^−2^**	**0.41 (0.24–0.69)**	**7.1 × 10^−4^**						
				Severe	0.81 (0.59–1.12)	2.0 × 10^−1^	0.92 (0.58–1.43)	7.0 × 10^−1^	0.71 (0.45–1.14)	1.6 × 10^−1^						
rs201563	PAX1 (20p11.22)	3′ Downstream	T	All	**1.37 (1.11–1.70)**	**3.2 × 10^−3^**	**1.49 (1.06–2.09)**	**2.1 × 10^−2^**	1.30 (0.99–1.71)	5.6 × 10^−2^	1.55	3.0 × 10^−81^	1.55	7.4 × 10^−82^	0.83	0
				Early	1.27 (0.97–1.66)	8.3 × 10^−2^	1.49 (0.97–2.30)	6.9 × 10^−2^	1.15 (0.81–1.62)	4.4 × 10^−1^						
				Severe	**1.50 (1.17–1.93)**	**1.3 × 10^−3^**	**1.59 (1.10–2.31)**	**1.5 × 10^−2^**	**1.44 (1.03–2.01)**	**3.2 × 10^−2^**						
rs2073963	HDAC9	Intron	G	All	**1.24 (1.02–1.51)**	**3.2 × 10^−2^**	0.94 (0.69–1.27)	6.8 × 10^−1^	**1.50 (1.16–1.94)**	**1.9 × 10^−3^**	1.29	1.0 × 10^−12^	1.27	3.6 × 10^−11^	0.05	74.66
				Early	1.20 (0.94–1.54)	1.4 × 10^−1^	0.94 (0.64–1.39)	7.7 × 10^−1^	**1.42 (1.03–1.96)**	**3.2 × 10^−2^**						
				Severe	**1.22 (0.96–1.53)**	**9.9 × 10^−2^**	0.93 (0.66–1.32)	6.9 × 10^−1^	**1.52 (1.11–2.09)**	**9.4 × 10^−3^**						
rs6047844	LINC01432	Intron	T	All	**1.34 (1.09–1.66)**	**6.6 × 10^−3^**	**1.48 (1.05–2.07)**	**2.4 × 10^−2^**	1.26 (0.96–1.66)	9.8 × 10^−2^	1.60	2.0 × 10^−39^	1.59	2.4 × 10^−39^	0.66	0
				Early	1.25 (0.95–1.63)	1.0 × 10^−1^	1.47 (0.95–2.26)	8.2 × 10^−2^	1.13 (0.80–1.6)	4.9 × 10^−1^						
				Severe	**1.47 (1.14–1.89)**	**2.6 × 10^−3^**	**1.59 (1.09–2.31)**	**1.6 × 10^−2^**	1.39 (0.99–1.94)	5.8 × 10^−2^						
rs1160312	LINC01432	Intron	A	All	**1.35 (1.09–1.67)**	**5.4 × 10^−3^**	**1.45 (1.04–2.03)**	**3.1 × 10^−2^**	1.29 (0.98–1.69)	7.0 × 10^−2^	1.60	1.0 × 10^−14^	1.58	1.3 × 10^−15^	0.59	0
				Early	1.24 (0.94–1.62)	1.3 × 10^−1^	1.41 (0.91–2.17)	1.2 × 10^−1^	1.14 (0.80–1.61)	4.7 × 10^−1^						
				Severe	**1.48 (1.16–1.90)**	**1.9 × 10^−3^**	**1.60 (1.10–2.32)**	**1.5 × 10^−2^**	**1.41 (1.01–1.96)**	**4.6 × 10^−2^**						
rs10888690	FAF1	Intron	C	All	**1.46 (1.08–1.97)**	**1.5 × 10^−2^**	1.43 (0.88–2.33)	1.5 × 10^−1^	1.47 (1.00–2.16)	5.1 × 10^−2^	1.11	6.0 × 10^−13^	1.11	1.6 × 10^−13^	0.31	4.81
				Early	1.41 (0.96–2.07)	7.8 × 10^−2^	1.20 (0.64–2.22)	5.7 × 10^−1^	1.55 (0.96–2.51)	7.2 × 10^−2^						
				Severe	1.41 (0.99–2.00)	5.8 × 10^−2^	1.53 (0.90–2.62)	1.2 × 10-1	1.32 (0.82–2.12)	2.5 × 10^−1^						
rs13021718	DPY30	Intron	A	All	**0.63 (0.46–0.86)**	**3.3 × 10^−3^**	**0.58 (0.36–0.94)**	**2.6 × 10^−2^**	**0.67 (0.45–0.99)**	**4.6 × 10^−2^**	0.81	2.0 × 10^−26^	0.81	2.0 × 10^−32^	0.18	43.81
				Early	0.73 (0.50–1.08)	1.2 × 10^−1^	0.77 (0.42–1.43)	4.2 × 10^−1^	0.71 (0.43–1.17)	1.8 × 10^−1^						
				Severe	**0.54 (0.36–0.80)**	**2.4 × 10^−3^**	**0.47 (0.26–0.84)**	**1.1 × 10^−2^**	**0.61 (0.36–1.04)**	**6.9 × 10^−2^**						
rs7976269	FAR2	5′ Upstream	A	All	1.23 (0.96–1.56)	9.6 × 10^−2^	**1.58 (1.07–2.33)**	**2.3 × 10^−2^**	1.04 (0.77–1.42)	8.0 × 10^−1^	1.15	6.0 × 10^−14^	1.15	2.4 × 10^−13^	0.11	60.58
				Early	1.15 (0.84–1.57)	3.8 × 10^−1^	1.22 (0.72–2.07)	4.6 × 10^−1^	1.11 (0.75–1.63)	6.1 × 10^−1^						
				Severe	1.29 (0.98–1.71)	6.9 × 10^−2^	**1.72 (1.14–2.61)**	**1.0 × 10^−2^**	1.00 (0.68–1.48)	9.8 × 10^−1^						

Note: SNPs, single nucleotide polymorphisms; A1, effect allele; early cases, early hair loss type (type 1 group of each of BASP classification L, M, C, and U); severe cases, severe hair loss type (type 2–3 group of each of L, M, C, and U); OR, odds ratio; CI, confidence interval; *p*, *p*-value; Q, Cochran’s Q; I, heterogeneity. Underline and bold results refer to enhanced significance in the meta-analysis *p*-values.

**Table 3:** Genome-wide significant and suggestive association results in each group.

CHR	BP	SNP	Mapped Gene	Function	A1	Effect Allele (A1) Frequency				Case Type	All Subjects		Males		Females	
						This Study	EAS	EUR	AMR		OR (95% CI)	*p*	OR (95% CI)	*p*	OR (95% CI)	*p*
**Significant in Both Sexes**																
8	142354673	rs7814359	TSNARE1	Missense p.(Phe18Leu)	G	0.34	0.35	0.20	0.29	All cases	**0.57 (0.46–0.69)**	**2.7 × 10^−8^**	**0.53 (0.39–0.72)**	**4.9 × 10^−5^**	**0.60 (0.46–0.78)**	**1.2 × 10^−4^**
										Early cases	**0.59 (0.45–0.76)**	**4.6 × 10^−5^**	**0.53 (0.36–0.79)**	**2.0 × 10^−3^**	**0.63 (0.45–0.88)**	**6.5 × 10^−3^**
										Severe cases	**0.54 (0.43–0.7)**	**1.4 × 10^−6^**	**0.52 (0.36–0.74)**	**3.1 × 10^−4^**	**0.57 (0.40–0.80)**	**1.1 × 10^−3^**
**Female Specific**																
7	91277819	rs2163085	FZD1	Flanking	C	0.25	0.22	0.36	0.46	All cases	**1.88 (1.50–2.35)**	**3.6 × 10^−8^**	1.45 (1.03–2.05)	3.5 × 10^−2^	**2.24 (1.67–3.01)**	**6.4 × 10^−8^**
										Early cases	**1.86 (1.40–2.47)**	**1.6 × 10^−5^**	**1.64 (1.05–2.56)**	**3.0 × 10^−2^**	**2.05 (1.42–2.95)**	**1.1 × 10^−4^**
										Severe cases	**1.74 (1.34–2.26)**	**2.8 × 10^−5^**	1.26 (0.86–1.85)	2.4 × 10^−1^	**2.24 (1.58–3.17)**	**5.4 × 10^−6^**
17	44823618	rs4793158	GJC1 (EFTUD2)	Intron	C	0.14	0.13	0.13	0.10	All cases	**2.15 (1.61–2.87)**	**1.9 × 10^−7^**	1.49 (0.95–2.34)	8.0 × 10^−2^	**2.70 (1.86–3.91)**	**1.5 × 10^−7^**
										Early cases	**2.38 (1.67–3.40)**	**1.6 × 10^−6^**	1.74 (0.98–3.09)	5.9 × 10^−2^	**2.88 (1.84–4.49)**	**3.4 × 10^−6^**
										Severe cases	**1.77 (1.26–2.48)**	**9.6 × 10^−4^**	1.21 (0.74–1.98)	4.5 × 10^−1^	**2.39 (1.53–3.75)**	**1.5 × 10^−4^**

Note. CHR, chromosome; BP, base pair; SNPs, single nucleotide polymorphisms; A1, effect allele; early cases, early hair loss type (type 1 group of each of BASP classification L, M, C, and U); severe cases, severe hair loss type (type 2–3 group of each of L, M, C, and U); OR, odds ratio; CI, confidence interval; *p*, *p*-value.

## Data Availability

The data used in this study can be shared after an internal review by e-mail request.

## Supplementary Materials

The following supporting information can be downloaded at: https://www.mdpi.com/article/10.3390/life14080939/s1, Figure S1: Example images of early cases and severe cases according to hair loss; Figure S2: Multi-tissue eQTL Plot; Table S1: Recapitulation results for previously AGA-associated SNPs.; Table S2: Lead SNPs of the suggestively or Significantly associated SNP region in all samples, male-only samples, and female-only samples.
