# Supplementary material for: Genetic Differences between Male and Female Pattern Hair Loss in a Korean Population
Source: Life (Basel). 2024 Jul 26;14(8):939. doi: 10.3390/life14080939 (PMC11355467; doi:10.3390/life14080939)
Supplement: Supplementary file 1 [file life-14-00939-s001.zip › life-3086365-supplementary/Figure S1.pdf]

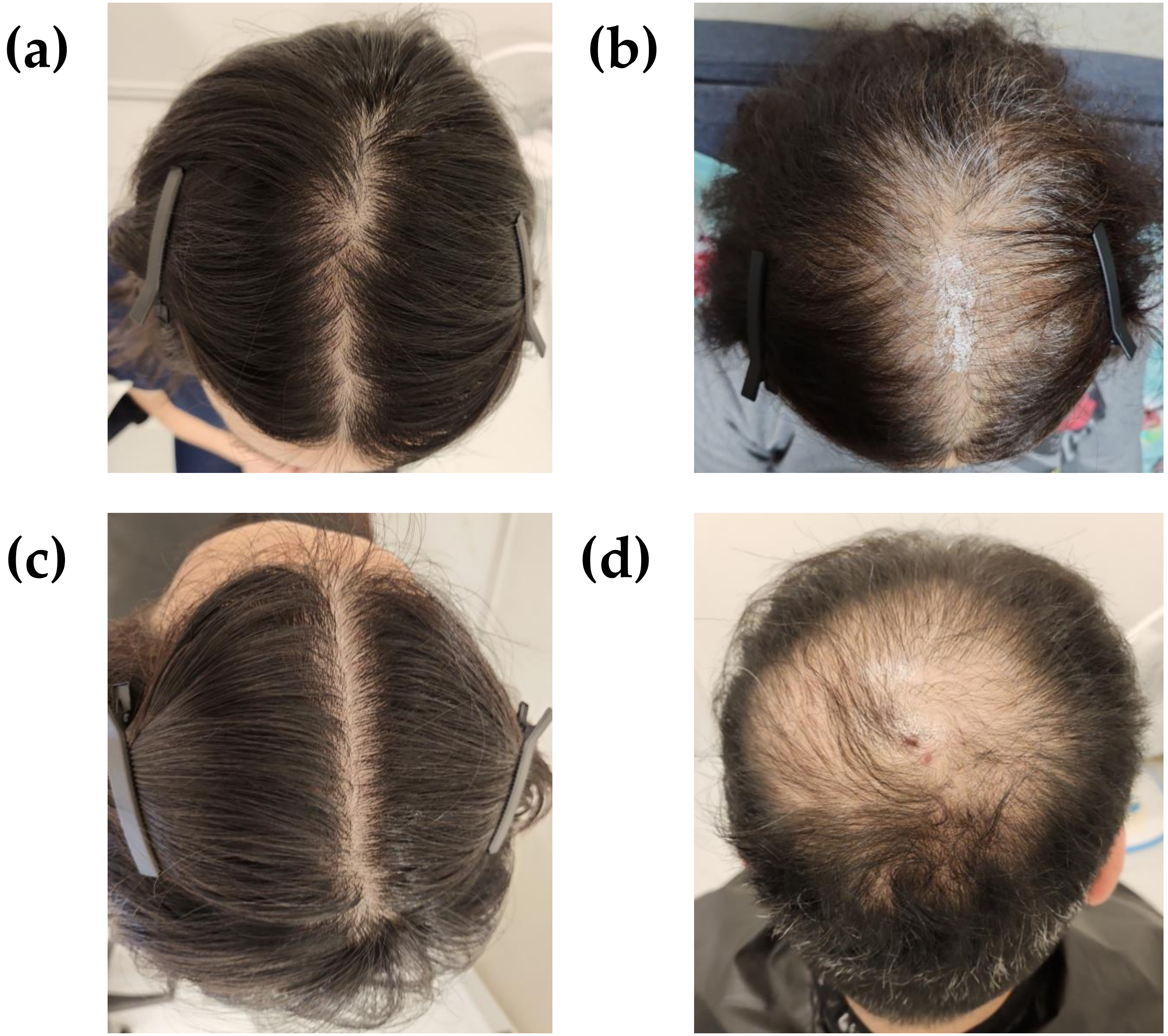

Figure S1. The example images of early cases and severe cases according to hair loss. (**a**) Early case in female; (**b**) Severe case in female; (**c**) Early case in male; (**d**) Severe case in male
