# Supplementary figures and images for: Genetic Differences between Male and Female Pattern Hair Loss in a Korean Population
Source: Life (Basel). 2024 Jul 26;14(8):939. doi: 10.3390/life14080939 (PMC11355467; doi:10.3390/life14080939)

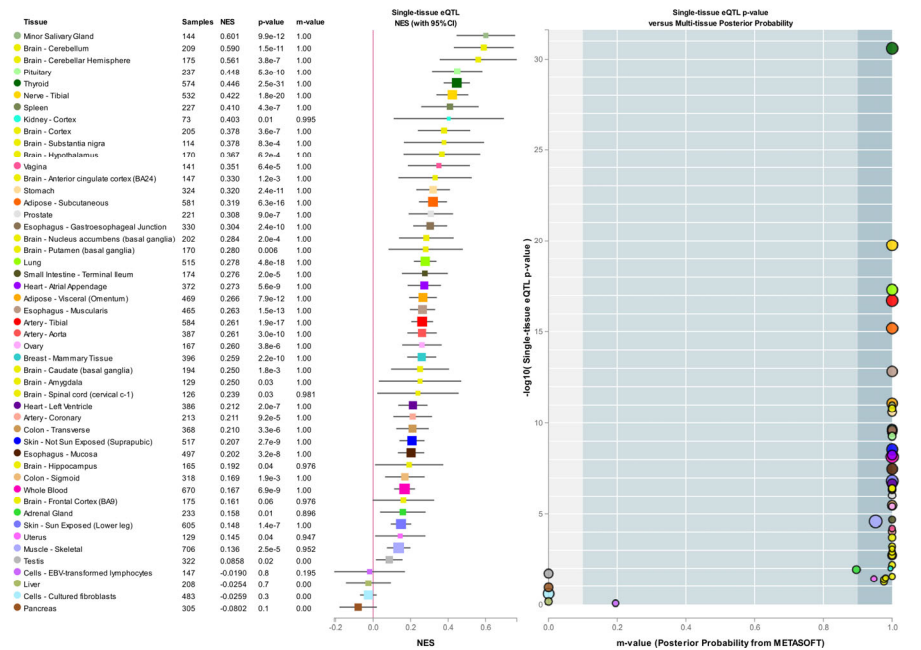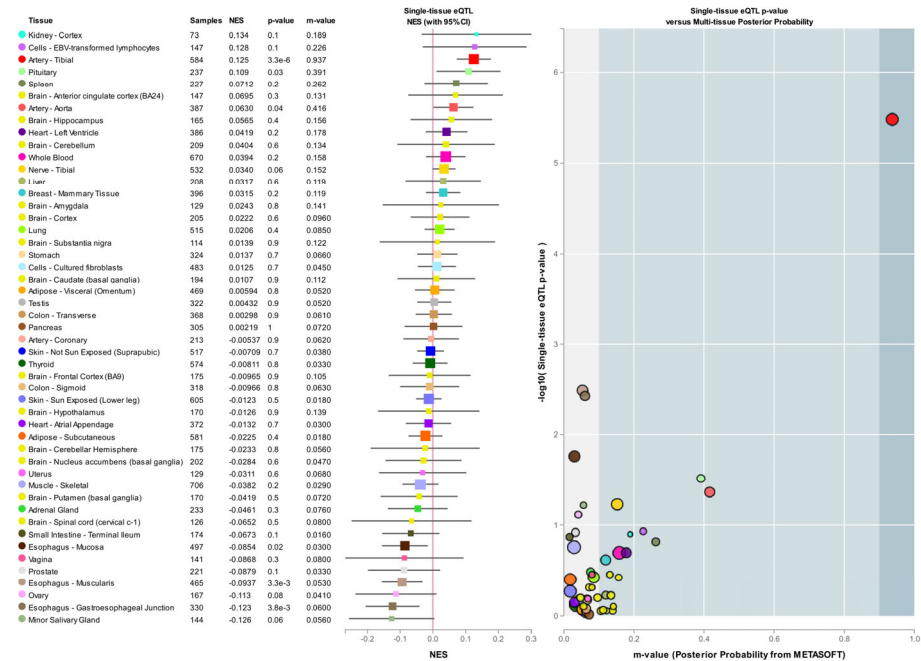

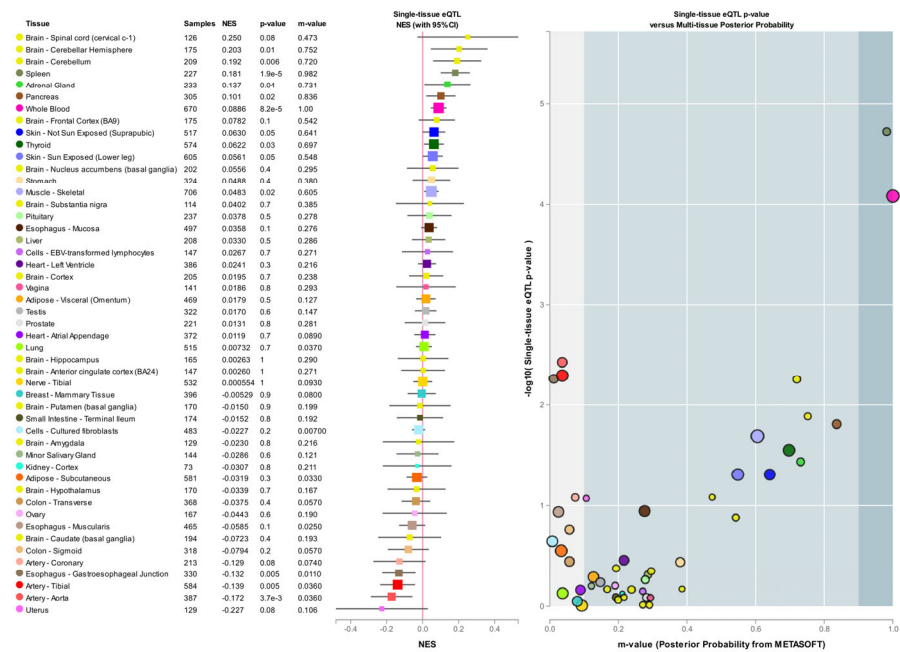

Figure S2: Multi-tissue eQTL Plot

Supplement: Supplementary file 1 [file life-14-00939-s001.zip › life-3086365-supplementary/Figure S2.pdf]
